# Muscle Hypertrophy in Prepubescent Tennis Players: A Segmentation MRI Study

**DOI:** 10.1371/journal.pone.0033622

**Published:** 2012-03-13

**Authors:** Joaquin Sanchis-Moysi, Fernando Idoate, Jose A. Serrano-Sanchez, Cecilia Dorado, Jose A. L. Calbet

**Affiliations:** 1 Department of Physical Education, University of Las Palmas de Gran Canaria, Las Palmas de Gran Canaria, Spain; 2 Department of Radiology, Clínica San Miguel, Pamplona, Spain; University of Rochester, United States of America

## Abstract

**Purpose:**

To asses if tennis at prepubertal age elicits the hypertrophy of dominant arm muscles.

**Methods:**

The volume of the muscles of both arms was determined using magnetic resonance imaging (MRI) in 7 male prepubertal tennis players (TP) and 7 non-active control subjects (CG) (mean age 11.0±0.8 years, Tanner 1–2).

**Results:**

TP had 13% greater total muscle volume in the dominant than in the contralateral arm. The magnitude of inter-arm asymmetry was greater in TP than in CG (13 vs 3%, P<0.001). The dominant arm of TP was 16% greater than the dominant arm of CG (P<0.01), whilst non-dominant arms had similar total muscle volumes in both groups (P = 0.25), after accounting for height as covariate. In TP, dominant deltoid (11%), forearm supinator (55%) and forearm flexors (21%) and extensors (25%) were hypertrophied compared to the contralateral arm (P<0.05). In CG, the dominant supinator muscle was bigger than its contralateral homonimous (63%, P<0.05).

**Conclusions:**

Tennis at prepubertal age is associated with marked hypertrophy of the dominant arm, leading to a marked level of asymmetry (+13%), much greater than observed in non-active controls (+3%). Therefore, tennis particpation at prepubertal age is associated with increased muscle volumes in dominant compared to the non-dominant arm, likely due to selectively hypertrophy of the loaded muscles.

## Introduction

There is discrepancy about training-induced muscle hypertrophy in preadolescents [1], [2], [3]. It has been suggested that inadequate levels of circulating androgens [3] or training stimulus [4] may limit muscle hypertrophy before puberty. Tennis is a good experimental approach to analyze whether exercise before puberty may elicit muscle hypertrophy [5], [6], [7], [8]. Both extremities have similar genetic endowment, are submitted to similar hormonal and nutritional influences, and therefore, side-to-side differences in arm muscle volumes reflect the maginitude of the exercise-induced muscle hypertrophy [8].

Using dual-energy X-ray absorptiometry (DXA), Calbet et al. [5] showed that male professional tennis players who started tennis practice before puberty had 20% more lean mass in the dominant than in the contralateral upper extremity. Recently, using the same method, it has been estimated that 50–75% of this asymmetry is attained at prepubertal ages [6]. A more detailed analysis using magnetic resonance imaging (MRI) found that in male professional tennis players dominant deltoid, triceps, arm flexors and forearm superficial flexors are hypertrophied 11–15% compared to the contralateral arm, whilst no significant differences were observed in the other muscle groups [8]. But the volumes of individual muscles have never been assesed with state-of-the-art technology (MRI) in prepubertal athletes. It remains to be determined whether tennis at prepubertal age elicits the hypertrophy of specific muscles of the dominant compared to the non-dominant arm using MRI.

Common injuries in tennis players have been associated to the asymmetric hypertrophy of the upper extremity, i.e., epicondilitis [9], [10] or shoulder impingement syndrome [11], [12], [13]. A better knowledge of the adaptability of the upper extremity muscles in prepubertal tennis players could advance in the undersatanding of the mechanisms leading to overload injuries in adulthood.

The main purpose of this study was to asses if participation in regular tennis training during prepuberty is associated with the hypertrophy of dominant arm muscles, and to determine which individual muscles of the dominant arm are specifically hypertrophied in response to tennis loading.

## Methods

### Subjects

Fourteen boys (age 11.0±0.8 y, Tanner 1–2) enrolled in the study. The participants were consecutively recruited from tennis clubs of Gran Canaria, as well as from one primary school through local announcements. To be included participants had to be below 12 years old, healthy, without any chronic disease and free of musculo-skeletal conditions or bone fractures. Seven of these boys were tennis players (TP) who had been participating in competitive tennis for a minimum of 2 years, with a frequency of at least 5 days per week. Control children (n = 7) were recruited from a primary school among children who did not participate in any regular form of exercise, apart from the compulsory physical education curriculum (2 weekly sessions of 45 min each), and were assigned to the control group (CG). Table 1 summarizes the main characteristics of each group.

**Table 1:** Physical characteristics and training history of tennis players and control group (mean ± SD).

Variables	Tennis (n = 7)	Controls (n = 7)
Age (years)	11.0±0.8	11.0±0.8
Tanner (1/2)	1/6	2/5
Height (cm)	146.7±6.0	146.0±5.0
Body mass (Kg)	37.4±6.2	43.8±5.4
**Tennis training history**		
Starting age	6.0±2.4	-
Current training volume (h/week)	11.6±2.2	-
Years playing	5.1±2.2	-
**Dominant arm/backhand stroke**		
Right/2 hands backhand	6/1	6/0
Left/2 hands backhand	1/1	1/0

Each participant and their parents were informed about the aims and procedures of the study and gave their informed signed consent to participate in the study. The study was approved by the ethical committee of the University of Las Palmas de Gran Canaria.

### Pubertal status assessment

Tanner pubertal status was self-assessed with parental guidance using the standard five scale Tanner stages [14].

### Magnetic resonance imaging

MRI was used to determine the muscle cross sectional area (CSA) and muscle volume of the arm and forearm muscles. A 1.5-T MRI scanner (Philips Achieva 1.5 Tesla system, Philips Healthcare, Best, The Netherlands) was used to acquire 10-mm axial contiguous slices from each arm independently, i.e., without interslice separation. Participants were positioned as described elsewhere [8]. Axial spin-echo T1-weighted MR images were acquired using a repetition time of 820 ms and echo time of 20 ms, with a 35-cm^2^ field of view and a matrix of 512×512 pixels (in plane spatial resolution 0.68×0.68 mm).

The acquired MRI images were transferred to a PC computer for digital reconstruction to determine the CSA. All calculations were carried out by the same investigator blinded to arm dominance using a specially designed image analysis software (SliceOmatic 4.3, Tomovision Inc, Montreal) for quantitative analysis of the images, as described elsewhere [15].

In the arms the volumes of the following muscles were assessed: the flexor compartment (*biceps brachii*, *brachialis* and *coracobrachialis*), *triceps* and *deltoid*. In the forearms we determined the muscle volumes of: *mobile wad* (*brachioradialis*, extensor *carpi radialis longus*, extensor *carpi radialis brevis*), flexors (*pronator teres*, flexor *carpi radialis*, flexor *carpi ulnaris*, *palmaris longus*, flexor *digitorum superficialis*, *pronator quadratus*, flexor *digitorum profundus*, flexor *pollicis longus*), extensors (extensor *carpi ulnaris*, extensor *digitorum communis*, extensor *digiti minimi*, *anconeus*, extensor *indicis propius*, extensor *pollicis longus*, extensor *pollicis brevis*, abductor *pollicis longus*) and *supinator* [16], [17]. To determine the distribution of muscle volume among muscles of a given tennis player, we calculated the volume fraction, expressed as a percentage of total muscle volume for each muscle, as described elswere [8], [18]. The mean volume fraction for each muscle across participants and volume fractions for the upper arm and forearm independently were also calculated.

### Statistical analysis

Mean and standard desviation of the mean are given as descriptive statistics in the text, standard error of the mean in the figures. Differences between groups were analyzed using a two factor mixed ANCOVA with side (dominant or non- dominant) as a within-subjects factor and group (TP or CG) as a between subjects factor, with height as covariate and post hoc differences tested with the Bonferroni test. Prior to the ANCOVA tests, variables were checked for normality using the Shapiro-Wilks test and for homogeneity with the Levene Test. Side-to-side differences into each group were assessed using Student's paired *t*-tests, adjusted for multiple comparisons with the Bonferroni-Holm method. The degree of asymmetry was calculated as the mean of the individual asymmetry values. Relationships between variables were assessed using the Pearson's correlation test. SPSS package (SPSS Inc., Chicago, IL, USA) for personal computers was used for the statistical analysis. Significant differences were assumed when P<0.05.

## Results

### Physical characteristics

The physical characteristics of the boys are summarized in Table 1. The groups were comparable in height, age and total body mass (P = 0.86, P = 0.79 and P = 0.06, respectively).

### Inter-arm asymmetry

**Figure 1:**
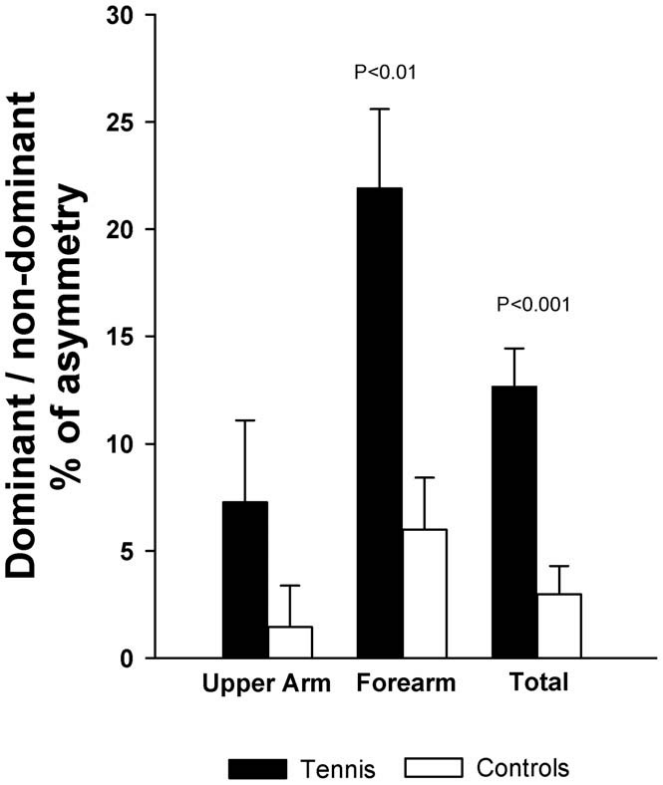
Differences in the degree of inter-arm asymmetry between prepubescent tennis players and non-active controls.

Table 2 summarizes total volume and muscle volume of each muscle group of the dominant and the non-dominant arms, in TP and control participants. Tennis players had greater total muscle volume in the dominant than in the contralateral arm (P<0.001), whilst no significant differences were observed in the CG (P = 0.07). The magnitude of inter-arm asymmetry was greater in TP than in controls (13 vs 3%, respectively, P<0.001) (Fig. 1).

**Table 2:** Muscle volumes of the dominant and non-dominant upper extremity (values expressed in cm^3^, mean ± SD) and asymmetries, comparisons are made between dominant and non-dominant sides into each group.

	Tennis Players				Controls			
Arm	Dominant	Non-dominant		ASY(%)	Dominant	Non-dominant		ASY(%)
Deltoid	155.2±21.0	139.1±25.1	*P<0.05*	**13**	136.6±18.1	128.2±14.6	*P = 0.30*	**7**
Triceps	162.2±16.1	170.3±41.6	*P = 0.60*	**−2**	166.7±13.8	170.7±17.2	*P = 0.29*	**−2**
Arm Flexor	165.8±35.1	144.7±35.0	*P = 0.27*	**19**	146.6±12.2	145.6±13.8	*P = 0.80*	**1**
Total arm	483.3±65.9	454.1±73.8	*P = 0.15*	**7**	449.8±38.6	444.5±43.8	*P = 0.54*	**1**

	Tennis Players				Controls			
Forearm	Dominant	Non-dominant		ASY(%)	Dominant	Non-dominant		ASY(%)
Flexors	149.5±18.2	125.2±17.9	*P<0.05*	**21**	150.4±14.7	143.0±14.6	*P = 0.14*	**5**
Extensors	58.7±4.4	48.0±7.7	*P<0.05*	**25**	48.5±3.4	49.7±5.6	*P = 0.55*	**−2**
Supinator	10.2±3.8	6.6±2.3	*P<0.01*	**55**	9.9±1.0	6.4±1.6	*P<0.05*	**63**
Mobile wad	125.9±60.1	103.2±49.6	*P = 0.20*	**29**	50.6±6.8	45.7±4.9	*P = 0.16*	**11**
Total forearm	344.3±45.3	282.9±33.1	*P<0.01*	**22**	259.4±21.2	244.9±17.6	*P<0.05*	**6**
**Total volume**	827.5±70.7	737.0±84.1	*P<0.001*	**13**	709.2±55.7	689.4±58.3	*P = 0.07*	**3**

**ASY:** Asymmetry between the dominant and non-dominant sides ((Dominant-Non-dominant)*100)/Non-dominant. The asymmetry represents the mean value of the individual asymmetries.

Compared to the non-dominant side, the dominant forearm had greater volume in TP and in controls (P<0.05). No significant differences were observed between both upper arms in TP and in CG. The magnitude of inter-arm asymmetry in the forearm was greater in TP than in CG (22 vs 6%, P<0.01). In the upper arms, the degree of bilateral asymmetry was similar in both groups (7 vs 1%, P = 0.20) (Fig. 1).

In TP, dominant forearm flexors, extensors and *supinator* muscles were hypertrophied compared to the contralateral side (P<0.05), whilst in the upper arm only the deltoid muscle volume was greater in the dominant compared to the non-dominant side (P<0.05). In controls, forearm *supinator* muscle was hypertrophied in the dominant compared to the contralateral side (P<0.05). In the other muscle groups, side-to-side muscle volumes were similar in TP, and also in controls. The magnitude of inter-arm asymmetry was significantly greater in TP than in CG in forearm extensors (P<0.05), whilst forearm flexors showed a trend to greater asymmetry in TP (P = 0.08). No significant differences were observed between TP and controls in the magnitude of asymmetry of *deltoid*, *triceps*, arm flexors, *supinators* and *mobile wad* (P = 0.20, P = 0.93, P = 0.17, P = 0.68 and P = 0.24, respectively).

The hypertrophied arm muscles maintained similar proportions between them, except for *supinator* forearm muscles which occupied a greater percentage of the total volume in the dominant than in the contralateral arm in both groups (P<0.05) (Table 3).

**Table 3:** Relative contribution of each muscle group to the total muscle volume of the dominant and the non-dominant upper extremity, in percentage (volume fraction), in prepubescent tennis players and normally active controls; * P<0.05, dominant compared to non-dominant arm.

	Tennis Players						
	Forearm muscle groups				Arm muscle groups		
	Flexors	Extensors	Mobile wad	Supinator	Deltoid	Arm flexors	Triceps
**Volume fraction dominant arm (%)**	18.1	7.2	15.1	1.2	18.8	18.2	19.6
**Volume fraction non-dominant arm (%)**	17.1	6.5	14.1	0.9*	18.9	17.1	23.0

	Controls						
	Forearm muscle groups				Arm muscle groups		
	Flexors	Extensors	Mobile wad	Supinator	Deltoid	Arm flexors	Triceps
**Volume fraction dominant arm (%)**	21.2	6.9	7.1	1.4	19.2	21.2	23.5
**Volume fraction non-dominant arm (%)**	20.8	7.2	6.7	1.0*	18.6	20.8	24.8

### Differences between groups

**Figure 2:**
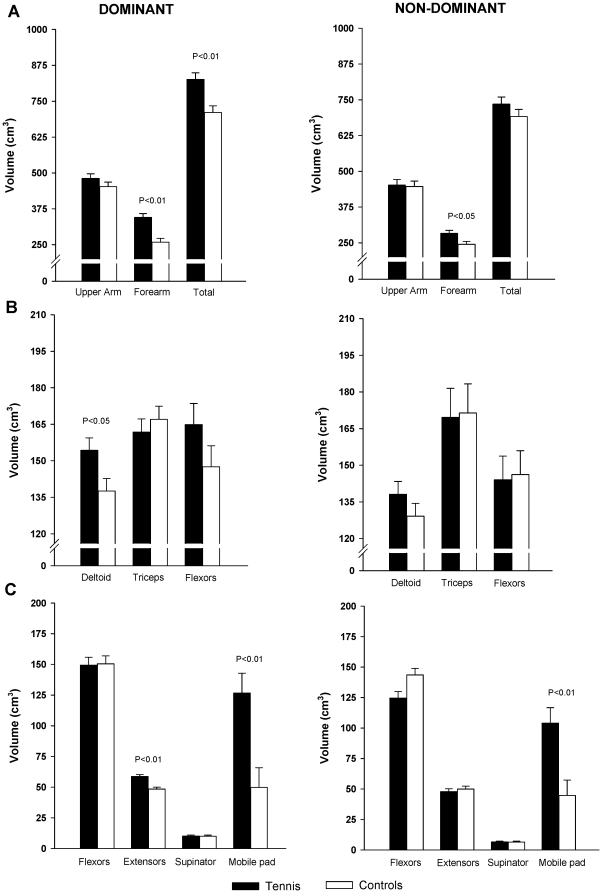
Muscle volumes of the dominant and non-dominant arms in prepubescent tennis players and non-active controls, after adjustment for height as covariate. (A) Total muscle volume of the arm, upper arm and forearm; (B) Volume of each muscle group of the upper arm and (C) of the forearm.

Total volume of the dominant arm was 14% greater in TP than in controls (P<0.01), whilst the non-dominant arm had similar volumes in both groups (7%, P = 0.24). Dominant (25%, P<0.01) and non-dominant (13%, p<0.05) forearms were greater in TP than in controls. After adjusting for height as covariate, total volume of the dominant arm was 16% (P<0.01) greater in TP than in controls, whilst the non-dominant arm had similar volumes in both groups (6%, P = 0.25) (Fig. 2). The dominant (33%, P<0.01) and non-dominant forearms (16%, P<0.05) had greater total volumes in TP than in controls. No significant between group differences were observed in the dominant and contralateral upper arms (7%, P = 0.28 and 2%, P = 0.77, respectively), neither after adjusting for height as covariate (6%, P = 0.23 and 1%, P = 0.86, respectively).

TP had greater volumes than controls in dominant forearm extensors (P<0.001) and *mobile wad* (P<0.05). Non-dominant *mobile wad* was also greater in TP than in CG (P<0.05). No significant between-group differences were observed in the other muscle groups of the dominant (P = 0.10, P = 0.59, P = 0.21, P = 0.92 and P = 0.84, for *deltoid*, *triceps*, arm flexors, forearm flexors and *supinator*, respectively) and contralateral arms (P = 0.34, P = 0.98, P = 0.95, P = 0.06, P = 0.63 and P = 0.89 for *deltoid*, *triceps*, arm flexors, forearm flexors, forearm extensors and *supinator* muscles, respectively). After adjusting for height as covariate, dominant *deltoid* was significantly greater in TP than in CG (P<0.05), whilst differences in dominant forearm extensors, and dominant and non-dominant *mobile wad* remained (P<0.01) (Fig. 2). After adjusting for height as covariate, no significant between-group differences were observed in the other muscle groups of the dominant (P = 0.50, P = 0.19, P = 0.90 and P = 0.93, *triceps*, arm flexors, forearm flexors and *supinator*, respectively) and contralateral arms (P = 0.26, P = 0.91, P = 0.88, P = 0.54, P = 0.05 and P = 0.97 for *deltoid*, *triceps*, arm flexors, forearm extensors, forearm flexors and *supinator* muscles, respectively).

## Discussion

The present study shows that tennis practice at prepubertal age is associated with 13% greater total muscle volume in the dominant compared to the contralateral arm. This asymmetry is significantly higher than the observed in the non-active control group (3%). The main contributors to the greater inter-arm asymmetry in TP were forearm muscles which were found to be 22% bigger in the dominant than in the contralateral side. The magnitude of inter-arm asymmetry observed in the TP of this study is similar to that reported in professional male tennis players using the same methods and equipment (12%) [8].

Our study shows that in prepubertal tennis players the muscles of the dominant arm have greater volume compared to the contralateral side. In addition, we observed that in TP the dominant arm had 16% greater volume than in children of comparable age and puberal status (Tanner ≤2), whereas non-dominant arms had similar volumes in both groups. Training-induced hypertrophy is the most likely mechanism to explain these findings. The effect of tennis on muscle size of the dominant arm observed in the present study is greater than the reported in healthy children using different strength training programs [1], [2], [3]. It is possible that the inclusion of plyometric movements in tennis strokes and not in the other studies [19], a higher training frequency in our TP (6 d/week) [6], together with a greater potential of the arms than the thighs for muscle hypertrophy [20], [21] may explain these differences. Fukunaga et al. [1], using ultrasound reported increased muscle CSA (7%) in the upper extremity of healthy boys, 9–10 years, following 12 weeks of maximal isometric strength training (3 d/week) [1]. In contrast, Ramsay et al. [3] using computerized tomography did not find a significant increase in the CSA of elbow flexors and knee extensors in prepubertal boys following 20 weeks of progresive resistance training (3 d/week). Analysing the lower extremities with MRI, Granacher at al. (2011) reported that 10 weeks of weight machines exercises did not change the quadriceps muscle CSA in children (9 years, circuit training, 2 d/week). Interestingly, a recent study using ultrasound conducted in peri-puberal boys and girls (7–17 years) with spastic cerebral palsy showed that the muscle volume of gastrocnemius increased a mean 15% in 5 weeks of progressive plantarflexor strengthening [22]. However, this results may not be comparable to the effects in healthy children because spastic cerebral palsy induce profound weakness of the muscles of lower limbs [23] with deficits in motor unit activation [24] and reductions in muscle volume [25], [26]. In addition, some participants were adolescents and the effects of the training program might have been influenced by the hormonal status [27].

Tennis strokes combine high intensity stretch-shortening cycles which have been demonstrated to be very efficient in eliciting muscle hypertrophy in adults [8]. In female prepubescent tennis players, Daly et al. (2004) reported 6.7% greater CSA in the dominant than in the non-dominant arm, but did not measure the muscle mass and did not include a control non-exercising group [28]. In male prepubescent tennis players, lean mass (DXA) was 10% greater in the dominant compared to the contralateral arm, and inter-arm asymmetry was greater in tennis players than in controls, however, between group differences in the lean mass of the dominant and non-dominant arms were not reported [7]. It has been estimated that 75% of the asymmetry in the lean mass of the arms observed in adult professional tennis players is attained at prepubertal age [6]. The present study shows that the increased dominant-arm muscle volume in prepubertal tennis players is similar to the reported in professional tennis players using the same MRI scanner (13 vs 12%, respectively) [8]. Therefore, our results indicate that high-intensity strength training may have a large potential to increase muscle mass in prepubescents [29].

Interarm asymmetry was significantly greater in TP than in controls in forearm muscles, but in the upper arms was similar in both groups. Dynamometric [30], [31] and electromyographic [32], [33] studies support a major role of forearm flexors and extensors during tennis strokes. Interestingly, the degree of asymmetry of forearm flexors observed in our TP is similar to that reported in professionals (both 13%) [8]. However, forearm extensors were more asymmetric in the children of the present study than in professionals (25 vs 5%, respectively) [8]. A possible explaination could be the greater relative strength developed by children to overcome the racket weight when swinging the racket during the preparation phase of the service and forehand strokes [33] and the acceleration phase of the backhand stroke [34], [35], [36], [37], particularly due to the lower ratio weight of racket/forearm muscle volume in children. In addition, eccentric contractions have a greater potential for muscle hypertrophy than concentric contractions [19]. It is well documented that during the backhand stroke less experienced players activate the forearm extensors eccentrically for longer time, whilst elite players activate these muscles concentrically and mainly during ball impact [34], [35], [36], [37]. On the other hand, in the upper arms, only *deltoids* were asymmetric in TP, but the degree of asymmetry was not significantly different than in CG. Interestingly, bilateral asymmetry of *deltoid* muscles in the TP was similar to the reported in professionals (13 vs 16%, respectively), whilst *triceps* and arm flexors were asymmetric in professionals but not in our prepubescent TP [8]. Although Sanchis Moysi et al. did not include a non-active group as a control [8], and it remains unknown what degree professional tennis practice contributes to the between-side *deltoid* muscle asymmetry in adults [38]. On the other hand, differences in *triceps* and arm flexors between prepubescents and professionals might be associated to the greater strength and power demands of tennis strokes in the professionals, especially during the serve [39], [40], or to different patterns of muscle recruitment.

Forearm muscles might have a greater potential for hypertrophy than upper arm muscles in children. Our results show that dominant and non-dominant *mobile wad* had greater volumes in TP than in controls, and that *mobile wad* was not asymmetric in any group. In the present study, only one TP used a two-handed backhand stroke and a similar effect on *mobile wad* was also observed when excluding this subject from the comparisons. Picking up tennis balls is a very repetitive movement performed bilaterally during tennis sessions which requires wrist extension because it is usually performed standing or squatting. Likely, bilateral hypertrophy of *mobile wad* in the TP, especially *extensor carpi radialis longus* and *brevis*, could be associated to this movement. On the other hand, *supinator* muscles were hypertrophied asymmetrically in TP and CG, and the magnitude of this asymmetry and the muscle volume of both arms were similar in both groups. This could indicate that daily activity performed by non-active boys was a sufficicient stimulus to elicit the hypertrophy of dominant *supinator*, regardless of tennis practice. However, aforementioned explanations are speculative and further studies are needed to analyse the potential of children for forearm muscle hypertrophy.

Epycondilitis or tennis elbow is a common injury in tennis players [41]. Isokinetic studies in adult tennis players have associated strength differences in forearm extensors and supinator muscles to injury [9], [10]. Future studies should investigate whether the asymmetric hypertrophy of forearm muscles observed in children may increase the risk of tennis elbow in adulthood.

In conclusion, tennis at prepubertal age is associated with marked enhancement of the muscle mass of the dominant arm, which achieves a total muscle volume that is 13% greater compared to the non-dominant arm. This asymmetry in arm's total muscle volume is greater than the 3% observed in non-active controls of comparable age and body size, and similar to the 12% asymmetry reported in adult professional tennis players [8]. Therefore, our study indicates that the skeletal muscle of prepubertal children has much greater plasticity than previously thought.
